# Efficacy and Safety of Lithium for Suicide and Suicide-Related Behaviors in Youth: A Review of the Literature

**DOI:** 10.3390/brainsci14111139

**Published:** 2024-11-13

**Authors:** Gianluca Sesso, Francesca Bargnesi, Francesca Olzi, Giulia Mutti, Stefano Berloffa, Valentina Viglione, Pamela Fantozzi, Greta Tolomei, Fulvio Guccione, Annarita Milone, Gabriele Masi

**Affiliations:** 1 IMT School for Advanced Studies, 55100 Lucca, Italy; gianluca.sesso@fsm.unipi.it; 2 Developmental Psychiatry and Psychopharmacology, IRCCS Stella Maris Foundation, 56128 Pisa, Italy; stefano.berloffa@fsm.unipi.it (S.B.); valentina.viglione@fsm.unipi.it (V.V.); pamela.fantozzi@fsm.unipi.it (P.F.); greta.tolomei@fsm.unipi.it (G.T.); fulvio.guccione@fsm.unipi.it (F.G.); annarita.milone@fsm.unipi.it (A.M.); 3 Department of Clinical and Experimental Medicine, University of Pisa, 56126 Pisa, Italy; francesca.bargnesi@fsm.unipi.it (F.B.); francesca.olzi@fsm.unipi.it (F.O.); giulia.mutti@fsm.unipi.it (G.M.)

**Keywords:** lithium, youth, adolescents, suicide

## Abstract

Objectives: This systematic review evaluates the anti-suicidal properties of Lithium in children and adolescents with Bipolar Disorder (BD), addressing gaps in evidence regarding its efficacy and safety in reducing suicidality and self-harming behaviors. Methods: A comprehensive literature search was conducted across PubMed, Web of Science, and Scopus up to February 2024. Eligible studies were those focusing on patients aged 25 years or younger, examining Lithium therapy and its impact on suicidal ideation and behaviors. The review included randomized controlled trials, longitudinal prospective and retrospective studies, and cross-sectional studies, while excluding expert opinions and case reports. Results: Evidence generally supports the efficacy of Lithium in reducing suicidal ideation and self-harming behaviors in youth with BD, though results are mixed. Randomized controlled trials demonstrated its effectiveness in mitigating suicidal thoughts during acute manic episodes, with effects persisting post-treatment. Longitudinal studies suggested that Lithium might offer superior outcomes compared to other mood stabilizers, although its specific impact on suicidality remains inconclusive. Cross-sectional studies and retrospective analyses reveal associations between Lithium use and reduced self-harming behaviors, but causality remains uncertain. While mood-stabilizing effects of Lithium offer potential benefits for reducing suicidality in youth, evidence on its direct impact on emotional dysregulation (ED) and long-term efficacy is limited. Variability in individual responses and adherence issues underscore the need for further research. Future studies should include larger, diverse samples, focus on ED symptoms, and explore Lithium mechanisms in suicidality prevention. Conclusions: Lithium remains a promising treatment for mood stabilization and reduction in suicidality in youth with BD.

## 1. Introduction

Lithium was the first medication proven effective for treating mood disorders and remains widely utilized for different psychiatric conditions in both adults and youths [1]. Its clinical efficacy has been demonstrated in managing mood disorders in adults, significantly alleviating both depressive and manic symptoms in Bipolar Disorder (BD) [2]. Lithium has also proven strong evidence in reducing suicide risk in adults [3,4,5], although recent meta-analyses questioned its anti-suicidal properties [6,7]. For children and adolescents, Lithium has received approval from major regulatory bodies, including the Food and Drug Administration and the European Medicines Agency, for treating BD [8]. Despite the high global prevalence of BD in youth, estimated at up to 3.4% [9], the evidence supporting Lithium efficacy in younger patients is less robust [10,11], particularly concerning its renowned effects on reducing suicidal and self-harming behaviors [12].

A critical review [1] reported a level 1b of evidence for Lithium on mania in pediatric BD, in line with the findings of two recent systematic reviews [13,14] suggesting that Lithium is an effective and well-tolerated treatment for some forms of pediatric mania. Less conclusive evidence is available for depression with suggestive studies providing a level 2b of evidence (e.g., [15,16]), while its monotherapy or augmentation use in bipolar depression is still controversial [17,18]. Additionally, Lithium also remains commonly used for different psychiatric conditions in youths, either or not in comorbidity with BD [1]. For instance, evidence is available for its use in Severe Mood Dysregulation [19], Conduct Disorder and aggression [20,21], Kleine–Levin Syndrome [22], and Fragile X Syndrome [23].

The pediatric transposition of the anti-suicidal properties of Lithium dates back in time. In 2008, Masters published an Editorial Letter [24] in response to Rosenberg and Salzman’s review on the new uses of Lithium in youth [25], which raises the issue regarding the role of Lithium Carbonate in the prevention of suicidality, based on practical evidence observed in a residential center for adolescents. Kept within plasma levels of 0.3–1.3 mg/mL, Lithium produced a prolonged remission of suicidal thoughts starting 48 h after the first dose and lasting until discharge in 59 patients with BD and comorbid Borderline Personality Disorder. Additionally, self-harming behaviors, in the form of self-cutting, also seemed to respond to the therapy, reducing in severity and frequency. More importantly, suicidal and self-harming behavior could recur 48–72 h after discontinuing the medication.

Interestingly, more recent longitudinal evidence tends to confirm such potential clinical benefits of Lithium in reducing or preventing suicide risk in young individuals with BD, though these findings are not yet comprehensive [26,27]. Similarly, while parasuicidal behaviors, including non-suicidal self-injury (NSSI), might also benefit from Lithium, the evidence remains sparse and is primarily derived from cross-sectional studies [28,29].

Theoretically, Lithium anti-suicidal effects may stem from its ability to reduce mood disorder relapses, though other mechanisms, particularly in youth, should be considered. Some evidence suggests that Lithium can decrease aggression and possibly impulsivity, which might also contribute to its anti-suicidal effects [3]. Indeed, it has been hypothesized that Lithium effectiveness in suicide prevention may be linked to its ability to diminish impulsive aggression within dysphoric, agitated, and mixed states, which are more prone to suicidal outcomes [30]. Furthermore, emotional dysregulation (ED), characterized by affective impulsivity and emotional reactivity, is increasingly recognized as a critical factor underlying suicidality within the developmental BD spectrum [31,32]. Unfortunately, direct evidence from randomized controlled trials or naturalistic studies on the efficacy of Lithium in addressing clinical measures of ED is still lacking, although there is a growing call for further research in this area to develop evidence-based guidelines for the use of Lithium in high-risk BD adolescents with ED symptoms and suicidality [28].

Despite all this evidence, Lithium anti-suicidal properties in youth are still controversial and clinicians cannot rely on consistent guidelines. Indeed, a discrepancy between evidence-based recommendations and clinical practice in using Lithium for young patients with BD has been highlighted [33]. Moreover, comprehensive reviews on the evidence available so far in youth are also missing. Thus, it is time to disseminate clear and unbiased information on the clinical efficacy of Lithium in BD patients to counteract the decreasing trend of prescribing one of the most effective drugs available for clinicians [33]. The aim of the present study was to systematically collect the literature evidence concerning the anti-suicidal properties of Lithium in the young population and to investigate the evidence for its efficacy and safety in preventing suicide and suicide-related behaviors in youth.

## 2. Materials and Methods

This systematic review was conducted across multiple databases, including PubMed, Web of Science and Scopus, up until 2 February 2024. There were no restrictions on language or document type. The search strategy was as follows: Lithium AND (Child* OR Adolescent* OR Youth* OR Young*) AND (Suicid* OR Self-Injury* OR Self-Cut* OR Parasuicid* OR Self-Harm OR NSSI). To be eligible for inclusion, studies had to meet the following criteria: (1) any article type except for expert opinions, editorial letters, and reviews; (2) any study design except for case reports or series (no restrictions related to the presence of a placebo or control group will be applied); (3) samples including only patients aged 25 years or younger; (4) any therapeutic formulation of Lithium salts; and (5) works that assessed any type of suicidal ideation and/or behavior and parasuicidal acts. Retrieved reviews were thoroughly assessed, as well as the bibliographies of eligible studies, in order to collect any further valuable study to be included.

Three authors (F.B., G.M., and F.O.) independently screened the titles and abstracts of all retrieved studies to identify those that potentially met the inclusion criteria. Any disagreements were resolved by consensus through discussion with a third author (G.S.). The full texts of articles deemed potentially eligible were then reviewed to confirm their inclusion. Data extraction from each study was qualitatively performed by three authors (F.B., G.M. and F.O.). Extracted data included study design, sample characteristics (such as sample size and age), clinical characteristics (such as clinical diagnoses and presence of co-occurring conditions), treatment details (such as Lithium formulation and dosage), and suicidality-related outcomes. The systematic review then led to a descriptive synthesis of all included studies. Studies were grouped according to the experimental design into four categories: (1) randomized controlled trials and open label studies; (2) longitudinal prospective studies; (3) longitudinal retrospective studies; (4) cross-sectional studies. A fifth category included studies in which suicidal outcomes were considered as a treatment-emergent adverse effect. No quantitative synthesis of the results has been reported since a meta-analysis could not be performed based on the retrieved studies. The Johns Hopkins Nursing Evidence-Based Practice (JHNEBP) rating scale was used to assess the quality of evidence of the included studies.

## 3. Results

### 3.1. Study Selection

**Figure 1:**
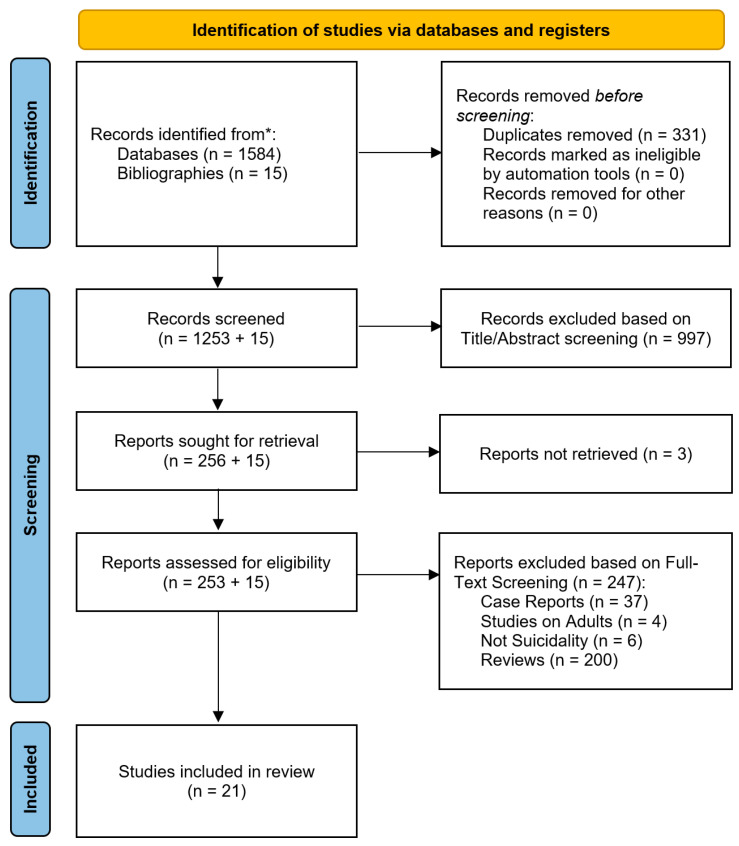
PRISMA flowchart. * Records were identified from three databases (PubMed, Web of Science and Scopus) and included studies’ bibliographies.

The flowchart in Figure 1 shows the identification and selection process of the papers. Out of the 1584 abstracts retrieved using our search strategy, 331 were removed as duplicates. Thus, 1253 studies were screened, of which 997 records were excluded based solely on title or abstract and 3 reports could not be retrieved. A total of 253 full-text articles were thoroughly assessed for eligibility. Based on a thorough assessment of reviews and the included studies’ bibliographies, fifteen additional records were identified and assessed for eligibility. Hence, 247 articles (47 original studies and 200 reviews) out of 268 were excluded (see Figure 1 for further details). Finally, 21 studies were included in the systematic review. The included studies are summarized in Table 1.

### 3.2. Randomized Controlled Trials and Open Label Studies

In 2003, Kafantaris and colleagues [34] conducted a large prospective open label study to examine the response and predictors of non-response to treatment with Lithium Carbonate in acute mania episodes in one hundred patients with BD type 1 aged 12 to 18 years. The treatment response was evaluated in terms of reduction in manic and depressive symptoms, suicidal ideation, and self-harming thoughts. Subjects received Lithium Carbonate rapidly titrated to reach the therapeutic levels (0.6 to 1.2 mEq/L) within the first week of treatment (mean plasma levels 0.93 ± 0.21 mEq/L; mean dosage 1355 ± 389 mg/day). Since treatment with other psychoactive drugs was not allowed, only 54 patients with Lithium monotherapy were included in the analysis, and treatment response was evaluated weekly. Findings from this study confirm the efficacy of Lithium in the acute stabilization of symptoms in moderate and severe acute mania episodes, and the presence of significant depressive symptoms did not diminish the response to therapy. Regarding suicide, a significant reduction in the percentage of patients with suicidal ideation, which was no longer reported in 82.61% of those who presented it at baseline, was described at the end of the 4-week treatment period.

Later on, in 2004, the authors followed up the same sample of patients and conducted the first placebo-controlled study of Lithium efficacy in the treatment of acute mania in adolescents [35]. In this discontinuation study, participants received open treatment with Lithium at therapeutic dosage (mean plasma levels 0.99 mEq/L) for at least 4 weeks. Responders were randomly assigned to either continue or discontinue Lithium during a 2-week, double-blind, placebo-controlled phase. Only subjects who met the response criteria—reduction in the Young Mania Rating Scale scores and the global improvement item of the Clinical Global Impression scale—at the end of the open treatment period were eligible to be randomly assigned to continue Lithium or to begin placebo for the 2-week double-blind phase. Importantly, 17 out of the 40 participants in the placebo-controlled discontinuation phase of this study (42.5%) had reported at least mild suicidal ideation at study entry. After open Lithium treatment, the mean score on the Hamilton Depression Rating Scale suicide item for this subgroup declined significantly from a moderate level of severity to nearly none. Of the seventeen participants with at least mild suicidal ideation at study entry, seven were assigned to continue Lithium treatment and ten were switched to placebo. During the double-blind phase, only three patients reported clear suicidal ideation, so they were removed from the study and the safety monitor broke the blind, revealing that all three had been on placebo and responded well to the resumption of Lithium treatment.

A brief but pertinent mention should be made of two important trials that have been conducted in the young population (see [3] for a review), although these studies’ objectives were not specifically aimed to assess the efficacy of Lithium for suicide and suicide-related behaviors. The first randomized trial was conducted by Findling et al. in 2005 [36] to determine whether Divalproex Sodium was superior to Lithium Carbonate in the maintenance monotherapy treatment of youths diagnosed with BD who had been previously stabilized on a combination therapy with both drugs. At the endpoint, Divalproex was not found to be superior to Lithium as a maintenance treatment, and the two groups did not differ in survival time until emerging symptoms of relapse or survival time until discontinuation for any reason. The second study was conducted by Pavuluri and colleagues in 2006 [37] to assess the safety and efficacy of Risperidone augmentation to Lithium in preschool-onset BD youths who insufficiently responded to Lithium monotherapy. The authors found that ADHD severity at baseline, a history of physical or sexual abuse, and preschool-age illness onset were significantly associated with an increased likelihood of poor response to Lithium monotherapy, and augmentation with Risperidone was safe and effective. Overall, no events of interest, including suicidal events, were reported in any of the two studies, although no specific assessment of suicidal ideation was available.

The TEAM (Treatment of Early Age Mania) study [38] was an 8-week, multicenter, single-blind RCT conducted by Geller and colleagues who compared the efficacy of Lithium Carbonate, Sodium Divalproex, and Risperidone on 279 antimanic drug-naïve children and adolescents aged 6–15 years old with BD type 1 on manic or mixed episode. Suicidal ideation was allowed in this sample if there was no imminent risk for suicide death. Patients were randomly assigned to receive either Lithium (initial dosage 150–300 mg twice per day titrated up to final doses, mean plasma levels 1.09 ± 0.34 mEq/L), Divalproex (mean plasma levels 113.6 ± 23.0 μg/mL), or Risperidone (mean dosage 2.57 ± 1.21 mg/day). In 2015, Salpekar and colleagues [16] conducted a secondary analysis on the TEAM study dataset focusing on the outcomes of Lithium, Divalproex, and Risperidone treatment in terms of depressive symptoms and suicidality. While depressive symptoms significantly reduced with faster improvements and lower dropout rates with Risperidone, suicidality—as assessed using the suicidal ideation item of the Children Depression Rating Scale—equally improved within the three groups from baseline to the endpoint with no significant differences between drugs. However, no significant effect could be identified specifically with any of the three drugs likely due to the low suicidality rate of the sample. The TEAM study contributions are also summarized by Robert Kowatch (see the review by the authors of [39]).

### 3.3. Longitudinal Prospective Studies

In 2003 and 2004, Jairam and colleagues [40,41] from India conducted a prospective follow-up study examining the course and outcome of juvenile-onset BD in a group of 25 patients aged 9 to 16 years treated with mood stabilizers, primarily Lithium, followed up for a period of 4 to 5 years. The study aimed to detect the rate and predictors of recovery and relapse of mood episodes, including mania with euphoria and/or grandiosity according to DSM-IV criteria. All patients received standard treatment comprising pharmacological and family counseling, and the former included mood stabilizers in monotherapy or in combination with antipsychotics. All patients started treatment with Lithium Carbonate with an average dose of 1074 mg/day (plasma levels 0.8 to 1.2 mEq/L). Four subjects did not respond to or could not tolerate Lithium; thus, Valproate and Carbamazepine were prescribed as alternatives, while four of the remaining 21 patients received Valproate in add-on. The prophylaxis period included therapy administration for at least 1–2 years after recovery from the index episode, with the possibility of reduction and discontinuation after a corresponding period of euthymia. Although all patients achieved remission of the index episode within the end of the study, 64% of patients had one or more relapses during the study period, of which 25.8% occurred when subjects were not taking medication and the remaining displayed Lithium plasma levels in the therapeutic levels, whereas 36% did not have any relapses. Moreover, one subject died by suicide by drowning, and two subjects attempted deliberate self-harm during relapse. The authors questioned the effectiveness of mood stabilizers, particularly Lithium, in preventing relapses and suicide-related events in young people. Nonetheless, it should be highlighted that the rate of relapses was 53% for the group compliant with the treatment and 100% for those who did not comply with the treatment, which clearly pleads for the usefulness of Lithium prophylaxis to reduce the overall number of relapses. Most clinical relapses, as reported by the authors, seem to have occurred in subjects with a poorer level of functioning, thus contributing to a high heterogeneity of the sample. 

In 2020, Hafeman and colleagues [26] published novel findings from the COBY study (Course and Outcome of Bipolar Youth), a naturalistic longitudinal prospective study on youths with BD aged between 7 and 18 years. The authors evaluated whether, compared to other mood-stabilizing drugs (OMSs), Lithium was associated with better outcomes in disease control, particularly concerning mood symptoms and suicidal tendencies. Clinical follow-ups were scheduled approximately every 6 months for an average total duration of 10 years, and 340 patients taking Lithium versus OMSs were monitored through clinical assessment focused on mood symptoms, suicidality, psychosocial functioning, hospitalizations rate, aggression, and substance use; weekly dosage of medications was also recorded. Standardized clinical tools including the Young Mania Rating Scale and the Children Depression Rating Scale were used to characterize mood symptoms. During the six-monthly follow-ups, the Longitudinal Interval Follow-up Assessment and the LIFE—Psychiatric Status Rating scales were used to evaluate weekly symptom fluctuations, as well as to evaluate suicidal tendency along with the previously mentioned ones. Since no data on the actual daily drug assumption were collected and plasma levels were not monitored weekly, Lithium exposure was based on weekly self reports and follow-up periods were classified as under Lithium or OMS whether patients reported to have assumed the drug for at least 75% of the weeks. After covariate adjustment, the Lithium group compared to the OMS group had half the suicide attempts rate, fewer depressive symptoms, less psychosocial deterioration, and less aggression.

### 3.4. Longitudinal Retrospective Studies

In 1987, Bashir and colleagues [42] conducted a retrospective observational study on 30 adolescents aged 13–17 years who were inpatients hospitalized and treated at the Rivendell Adolescent Sydney Center between 1972 and 1982. Patients were included in the study if they had presented manic or hypomanic symptoms on at least one occasion and were followed up for at least 6 months after discharge. Pharmacological therapy, supported by psychotherapy, was the most frequently employed treatment modality. During hospitalization, most subjects were initially treated with antipsychotic agents, while Lithium Carbonate was used to control affective symptoms and was the preferred treatment, especially in the presence of hypomania or mania and when relapses were rapid or frequent despite antipsychotic therapy. Lithium plasma levels at discharge were 1.0 mmol/L on average (range 0.8–1.2 mmol/L) and were reached and then maintained for nine to twelve months, after which decision to continue or discontinue therapy was based on affective stability and compliance. At discharge, one-third of the subjects were not taking any medication and one-third were on Lithium as maintenance therapy, of which 10% were on Lithium alone and half were taking antipsychotics, mainly for agitation control, with a minority continuing to take antidepressants. At the last contact, one-fifth of the patients did not need to take psychotropic drugs. At least one-third of the patients made one or more suicide attempts during the course of the illness, but no deaths from complete suicide occurred during the ten years of the study and the three years following, especially among BD subjects. 

In 2008, Jerrell [29] conducted a retrospective cohort study over an 18-month follow-up period, during which the medical records of 82 patients, aged between 6 and 18 years, with a diagnosis of BD type 1, hospitalized in a public mental health hospital between 2003 and 2004, were examined. The study examined the services, medications received, and changes in psychosocial functioning over time. More than 60% of the subjects were prescribed mood stabilizers, of which 4.9% were on Lithium Carbonate at an average dose of 900 mg/day (daily dosage range 600–900 mg); other psychotropic medications were also administered in combination. Combined treatment with mood stabilizers and SGA was associated with a decrease in self-harming, anxious, and depressive symptoms; however, this finding was difficult to attribute directly to pharmacotherapy in this small cohort, also considering the multidisciplinary treatment approach. 

In 2018, our research group published a naturalistic retrospective study [43] based on a clinical dataset including 30 patients aged 14.2 ± 2.1 years to describe the safety and efficacy of Lithium Carbonate in referred BD adolescents who were followed up for eight months of treatment. Mean Lithium dosage was 843 ± 176.6 mg/day after 4 months (T1) and 858 ± 149 mg/day after 8 months (T2); mean blood level of Lithium was 0.69 ± 0.20 mEq/L at T1 and 0.70 ± 0.18 mEq/L at T2. Overall, Lithium treatment—alone or in combination with other psychotropic drugs—significantly improved clinical severity and functional impairment in the first four months, and this was confirmed in the following four months without relapses. Emergence of both suicidal ideation and behavior was not reported during the follow-up. Although suicidality was not among the primary targets of the study, safety was generally preserved, and the rate of side effects was lower compared to similar studies in adult populations, likely due to relatively low blood levels in the sample. 

Later on, in 2020, our research group published another naturalistic retrospective study [44] based on a clinical database of youths with BD consecutively referred during a 2-year period (2016–2018) for manic or hypomanic symptoms and followed up for at least 6 months. The aim of the study was to explore the effect of gender and age at onset (prepubertal versus adolescent onset) of BD as well as comorbid ADHD and substance use disorder on response to treatments and to explore possible features associated with severe suicidal ideation or attempts with poorer response to treatments. The sample consisted of 117 patients aged between 7 and 18 years, with a mean age at the time of admission of 14.5 ± 2.6 years. All patients received a pharmacological treatment according to clinical guidelines: BD patients were primarily treated in monotherapy with a mood stabilizer, including Lithium or Valproic Acid, and then with an association of both drugs if severely ill, while SGAs were used in non-responders or when psychotic symptoms and/or severe aggression and hostility were associated. Other medications, including Methylphenidate and SSRIs, were used when needed. In the sample, 81.2% of the patients received a mood stabilizer, only 18.8% received a first SGA monotherapy, and only 38.5% remained on a mood stabilizer monotherapy, while a strong minority (42.7%) received a polypharmacy with both a mood stabilizer and SGAs. Twenty-five patients (21.4%) presented severe suicidal ideation or behavior, but none of the selected measures differentiated patients with or without suicidality, including age at onset of BD, anxiety, ADHD, or SUD comorbidities and treatments. It may be possible that a larger sample may support the possible role of specific clinical features. 

In 2023, Desai Boström and colleagues [27] published the results of an analysis conducted using data from the Swedish national registers between 2016 and 2020. The sample included 200 confirmed suicide deaths among young patients aged 15 to 19 years and the analysis retrospectively investigated the association between adolescent suicide mortality and the use of Lithium, Clozapine, and Electroconvulsive therapy (ECT). The results highlighted a negative correlation between suicide mortality and frequencies of Lithium, Clozapine, and ECT use among adolescents, which was confirmed for males but not for females. In regions with the lowest suicide mortality rate, higher Lithium use was observed, as well as a higher negative correlation with Lithium, Clozapine, and Electroconvulsive therapy in males. Post hoc analyses of individual treatments confirmed associations between the regional use of Lithium in adolescents and a lower suicide mortality rate, while ECT and Clozapine use rates in adolescents did not show a significant trend. The authors concluded that early recognition and Lithium treatment in male adolescent patients with severe mental disorders may be useful in reducing the risk of suicide mortality.

### 3.5. Cross-Sectional Studies

Ko et al. in 2014 [28] conducted a cross-sectional study on 100 adolescents aged 13 to 19 years with BD type 1 or 2 or not otherwise specified, recruited consecutively at a tertiary subspecialty clinic for adolescent BD in Ontario. The study examined factors associated with Lithium use among adolescents with BD. Only 20% of participants reported lifetime use of Lithium. Those who had used Lithium in their lifetime, compared to those who had not, were significantly older and more likely to have BD-I, a history of psychiatric hospitalization, and psychosis. They also reported a higher use of second-generation antipsychotics and anticonvulsant mood stabilizers. Conversely, participants exposed to Lithium had a significantly lower likelihood of having BD-II, engaging in self-harming behaviors, and having a family history of depression, family conflicts, and parent-reported mood lability. They also reported significantly less impulsivity, emotional dysregulation, identity confusion, and interpersonal problems. Suicidal ideation was not significantly associated with Lithium exposure, but its prevalence was lower among those with lifetime Lithium use compared to those without. The lower rates of self-injurious behavior and suicidal ideation among Lithium-exposed participants might be a direct effect of Lithium treatment or be related to clinicians’ reluctance to prescribe Lithium to adolescents due to its lethality in overdose situations. Despite this, along with a trend towards a higher prevalence of a family history of suicide attempts among adolescents without Lithium exposure, these results suggest the general conclusion that Lithium appears to be underutilized among adolescents with BD who are at clinical and/or familial risk for suicide. 

Otuyelu et al. in 2015 [45] analyzed the relationship between the use of antidepressants and Lithium and the suicide rate among youths under 20 years old in Hungary through a cross-sectional design study conducted between 1998 and 2006. Mortality data were sourced from the Hungarian Central Statistical Office, while data on drug prescriptions were obtained from the Hungarian National Health Insurance Fund. Overall, 479 recorded deaths by suicide (375 males and 104 females) were collected. Descriptive data highlighted a consistent increase in the prescription of Lithium and different antidepressants, which was about 8-fold in the study period, while the suicide rate among individuals under 20 years decreased by 11%. A generally significant inverse association was found between the temporal trends of antidepressant and Lithium use and suicide rates, and this association was also significant individually for the prescription of Lithium, SSRIs, and other antidepressants, but not MAOs and non-selective monoamine reuptake inhibitors, suggesting that these drugs might play a role in reducing suicide-related deaths in adolescents. Albeit interesting, these findings suffer from some major limitations. The cross-section design of this ecological study cannot infer causality at an individual level; thus, it does not allow for the examination of temporal relationships of these associations. Secondly, besides the fact that health insurance is mandatory for every citizen living in Hungary, this suggests that we can safely assume that the antidepressant prescription data from the Hungarian National Health Insurance Fund—which has the personal identification and prescriptions of all insured persons—gives a true representation of the national drug prescription, we cannot be certain that it can be uniquely referred to the Hungarian Central Statistical Office mortality data. 

Janiri et al. in 2021 [46] conducted a cross-sectional study to investigate the impact of emotional dysregulation and temperamental features on suicidality in a sample of fifty young patients aged 14–25 years with mood disorders from a University Hospital in Rome, along with a matched sample of healthy controls. Among the patients, 48% reported having had suicidal ideation. In the patient group enriched with suicidal ideation, there was a higher familial history of psychiatric disorders, a greater use of Lithium and antipsychotic medications, higher levels of emotional dysregulation, and a greater endorsement of cyclothymic temperament compared to patients without suicidal ideation. Additionally, the majority of participants in the suicidal ideation group reported receiving psychotherapy. Two correlational hypotheses can be inferred from these findings. First, patients with a greater severity of illness, who require the use of Lithium, are also those at greater risk of suicidal ideation; alternatively, Lithium could have been prescribed to these patients precisely because of the presence of suicidal ideation, as part of a treatment aimed at stabilizing mood and reducing the risk of suicide. Either way, this underscores the importance of addressing emotional dysregulation and temperament in the assessment and treatment of suicide risk in young patients with mood disorders.

In 2023, Andersson and colleagues [47] conducted a cross-sectional study with retrospective observation on the association between adolescents’ suicide mortality, stratified by sex, the frequency of BD diagnoses, and the use of Lithium. Data were retrieved from the National Registries across 21 Swedish regions in the period from 2008 to 2021 and included all registered Swedish citizens aged 15 to 19 years who died by suicide and/or had been diagnosed with BD stratified by region and sex. The sample consisted of 585 unique observations of confirmed suicide deaths and the main outcomes were sex-stratified, regional, annual suicide mortality rates in adolescents aged 15 to 19 years per 100,000 inhabitants analyzed using generalized linear mixed-effects models. The authors found that regions with higher rates of BD diagnoses among male adolescents also prescribed Lithium to a higher number of patients, although with higher discontinuation rates. This could indicate that male adolescents may discontinue Lithium treatment because of possible adverse effects or other issues with adherence to medication. The frequency of BD diagnoses was strongly associated with a decrease in adolescent male suicide mortality rates, estimated to reduce the national average by about 4.7%. However, notably, this association remained significant regardless of the regional annual number of Lithium dispensations, which showed a non-significant trend in the same direction. These findings indicate that regional factors associated with the diagnosis of BD may exert protective effects against suicide in male adolescents which appear to be unrelated to Lithium prescription. Conversely, in females, while BD diagnosis frequency and Lithium treatment utilization rates were not associated with suicide death rates, an independent positive association was observed between BD frequency and Lithium prescription rates. 

### 3.6. Suicide as an Adverse Effect

The first released article from the TEAM study described above was published in 2012 [38]. In this paper, the authors reported that Risperidone resulted to be significantly more effective on CGI—Bipolar score improvement than both Lithium and Divalproex in the initial management of mania in children, while less tolerated with higher rates of metabolic effects. Dropout rate was higher for Lithium than Risperidone, and 7 patients displayed severe suicidal behaviors; however, none of them was considered to be an adverse effect of the treatment. 

In 2015, Findling and colleagues [10] conducted a multicentric, double-blind, placebo-controlled RCT on 153 young patients aged 7 to 17 years old with BD type 1 during manic or mixed episodes to assess the effectiveness of Lithium acute administration over 8 weeks of treatment. Patients were randomized to either Lithium (initial dosage 600–900 mg/day, mean dosage 1292 ± 420 mg/day for 7–11 years and 1716 ± 606 mg/day for 12–17, mean plasma levels 0.98 ± 0.47 mEq/L) or placebo based on a 2:1 allocation ratio. While Lithium was found to be superior to placebo in reducing manic symptoms and well tolerated, especially in terms of weight gain, two participants on Lithium and one on placebo withdrew from the study due to increased suicidality. It should be noted, however, that the presence of severe suicidal thoughts and related manifestations was listed among the exclusionary criteria of the study; nonetheless, the increased suicidality observed in the two patients on Lithium was not considered to be associated with the ongoing treatment. 

A recently published 6-week double-blind RCT by Patino and coworkers [48] investigated the efficacy of Lithium and Quetiapine for the treatment of manic/mixed episodes in 109 early-onset BD adolescents aged 10 to 17 years. Patients were randomized to either Lithium (mean dosage 1023 ± 362 mg/day, mean plasma levels 0.57 ± 0.4 mEq/L) or Quetiapine (mean dosage 428.3 ± 100 mg/day). Both agents resulted to be effective in reducing manic symptoms and achieve remission, with Quetiapine exhibiting faster and larger, although marginally, response effects. Suicidal ideation and behavior were assessed at every visit using the Columbia Suicide Severity Rating Scale among the tolerability measures. Both drugs displayed good tolerability in terms of suicidality.

In 2011, Findling and colleagues [49] conducted an exploratory study to obtain data that could lead to evidence-based dosing strategies for Lithium in children and adolescents suffering from BD type 1 (either manic or mixed episode). Fifty-seven outpatients aged 7–17 years were enrolled for an 8-week open label trial with Lithium Carbonate in one of three dosing arms. The mean total daily dose was 1500 ± 400 mg, whereas the mean weight-adjusted total daily dose was 29.1 ± 8.0 mg/kg/day. The mean serum concentration at the end of the treatment was 1.05 ± 0.39 mEq/L. A priori response criteria were defined using the Clinical Global Impression—Improvement scale and the Young Mania Rating Scale. The Suicide Severity Rating Scale was also completed weekly. Most patients had a 50% improvement in manic symptoms, and more than half of the patients achieved a full response. Lithium was well tolerated, and all three treatment arms had similar effectiveness, side effect profiles, and tolerability of Lithium. Serious TEAEs were experienced by a total of six patients (10.0%), only one of which displayed suicidal ideation which was considered to be possibly or probably related to Lithium. 

**Table 1:** Summary of included studies.

Authors	Year	Design	JHNEBP	SoE	QoE	Sample	Age	Findings	Limitations
Bashir et al.	1987 [42]	Retrospective observational study	NES	III	Low	30	13–17	1/3 patients made ≥1 suicide attempt during illness but no complete suicide during 13 years of study among BD	Only in 5 cases was Lithium the only maintenance therapy
Kafantaris et al.	2003 [34]	Prospective open label study	NES	III	Low	100	12–18	Significant reduction in rate of suicidal ideation, no longer reported in 83% of those who presented it at baseline	Add-on antipsychotics in 46% of sample, especially in those with psychosis or severe aggression
Jairam et al.	2003, 2004 [40,41]	Longitudinal prospective follow-up study	NES	III	Low	25	9–16	Low rates of suicide attempts and completed suicides in those under Lithium therapy	Small sample size, uncontrolled treatment and low comorbidity rate in the sample
Kafantaris et al.	2004 [35]	Placebo-controlled study	ES—RCT	I	Good	40	12–18	During double-blind phase 3 patients reported clear suicidal ideation, but were on placebo and responded well to the resumption of Lithium	Higher than expected rate of exacerbation, outpatients only, discontinuation design as indirect assessment of Lithium efficacy
Findling et al.	2005 [36]	Double-blind 18-month RCT	ES—RCT	I	Good	60	5–17	Divalproex not found to be superior to Lithium as maintenance therapy; the two groups did not differ in survival time	Small sample size, no placebo controlled, possible nocebo effect
Pavuluri et al.	2006 [37]	Open label trial	NES	III	Low	38	4–17	ADHD severity, history of physical/sexual abuse, preschool-age illness onset associated with poor response to Lithium monotherapy, add-on with Risperidone safe and effective	Small sample size, no randomization to add-on with Risperidone
Jerrell et al.	2008 [29]	Retrospective cohort study	NES	III	Low	82	6–18	Combined treatment with mood stabilizers and SGA associated with decreased self-harm, suicidality, anxiety, and depression	Only 4.9% on Lithium, thus difficult to directly attribute the effects to pharmacotherapy
Findling et al.	2011 [49]	Open label trial	NES	III	Low	57	7–17	Lithium well tolerated, and all three dosing treatment arms with similar effectiveness, side effect profiles, and tolerability, only one patient with suicidal ideation	Lithium levels not fully accurate, no efficacy assessment of maintenance therapy
Geller et al.	2012 [38]	Single-blind RCT	ES—RCT	I	Good	279	6–15	Risperidone more effective than Lithium and Divalproex but with severe metabolic effects, suicidality significantly decreased for all medications with 7 suicidal behaviors	Too few non-psychotic subjects for meaningful analyses of this subgroup
Ko et al.	2014 [28]	Cross-sectional study	NES	III	Low	100	13–19	Suicidal ideation not significantly associated with Lithium exposure, but lower rates of self-injurious behavior and suicidal ideation in those with Lithium exposure and higher prevalence of family history of suicide attempt in those without	Only 20 participants exposed to Lithium; no available data on dosage, plasma levels, adherence, and duration of treatment; exposure determined via clinical interview
Salpekar et al.	2015 [16]	Prospective randomized clinical trial	ES—RCT	I	Good	279	6–15	Suicidality equally improved within the three treatment groups with no significant differences between Lithium, Divalproex, and Risperidone, but no significant effect identified due to low suicidality rate	Secondary analysis, possible missing data due to dropouts
Findling et al.	2015 [10]	Double-blind, placebo-controlled RCT	ES—RCT	I	Good	153	7–17	2 patients on Lithium and 1 on placebo withdrawn due to increased suicidality, but not considered to be associated with the ongoing treatment	Brief study period, severe suicidal ideation listed as exclusionary criteria
Otuyelu et al.	2015 [45]	Cross-sectional study	NES	III	Low	479	<20	Inverse association between temporal trends of antidepressant and Lithium use and suicide rates, also significant individually for the prescription of Lithium and antidepressants	Mortality data from the national registries
Masi et al.	2018 [43]	Naturalistic retrospective study	NES	III	Low	30	12–18	Emergence of suicidal ideation and behavior not reported during follow-up	Data gathered only from patients who completed follow-up, many patients with other medications
Hafeman et al.	2020 [26]	Longitudinal Prospective Study	QES	II	Good	340	7–18	Lithium group with half the suicide attempt rate compared to other mood stabilizers	No collected data on daily dose or blood levels, nor on side effects or tolerability, possible underestimation of suicidal ideation
Masi et al.	2020 [44]	Naturalistic retrospective study	NES	III	Low	117	7–18	21% of patients presented severe suicidal ideation or behavior, but none of the measures differentiated those with or without suicidality including treatment	Only highly comorbid and severely impaired patients, brief period of observation
Janiri et al.	2021 [46]	Cross-sectional study	QES	II	Good	50	14–25	48% with suicidal ideation associated with greater use of Lithium or antipsychotic, higher emotional dysregulation, and cyclothymic temperament compared to patients without	No quantitative measure of suicidal risk; no stratification based on mood disorder
Patino et al.	2021 [48]	Double-blind RCT	ES—RCT	I	Good	109	10–17	Lithium and Quetiapine with good tolerability in terms of suicidality, assessed at every visit using Columbia Suicide Severity Rating Scale	Higher drop off rate in the Lithium group possibly due to slower response and different tolerability
Desai Boström et al.	2023 [27]	Cross-sectional study	NES	III	Low	200	15–19	Lower suicide mortality rate associated with higher Lithium use and negative correlation with Lithium, Clozapine, and Electroconvulsive therapy in males	Males generally over-represented, assessing excess suicide rates as opposed to baseline values, and biological differences between genders
Andersson et al.	2023 [47]	Cross-sectional study	NES	III	Low	585	15–19	Regions with higher rates of BD among male adolescents with higher Lithium prescription rates although with higher discontinuation rates; rates of BD also strongly associated with decreased adolescent male suicide mortality	No significant association in females due to potential over-diagnosis of BD in female adolescents

## 4. Discussion

The present systematic review aims to address a critical gap in the literature by examining the anti-suicidal properties of Lithium in the young population. Lithium, historically recognized for its efficacy in treating mood disorders, remains a cornerstone in the management of Bipolar Disorder. Despite its established role in adults, its effectiveness in youth, particularly in reducing suicidality, has been less clear and comprehensive reviews on the evidence available so far in youth are also missing. This review synthesizes available data on Lithium impact on suicidal behaviors in adolescents, offering a nuanced perspective on its clinical utility. The main contribution of our review is to disseminate clear and unbiased information on the clinical efficacy of Lithium for suicide prevention in young BD patients.

The review identified twenty-one studies encompassing randomized controlled trials, longitudinal prospective studies, retrospective cohort studies, and cross-sectional studies. The evidence reflects a mixed but generally supportive view of Lithium efficacy in reducing suicidal ideation and behaviors in youth with BD. Among the available RCTs, the study by Kafantaris et al. [34] and its follow-up [35] highlight Lithium effectiveness in reducing suicidal ideation among adolescents with BD during acute manic episodes. Significant improvements were noted, and the reduction in suicidal thoughts persisted post-treatment. However, the study duration and sample size limit the generalizability of these findings. Other trials, such as those by Findling et al. [36] and Pavuluri et al. [37], though not specifically focused on suicidality, suggest that Lithium remains comparable to other treatments in maintaining mood stabilization and preventing relapses. The TEAM study [38] indicated that, while Lithium improved mood symptoms, its impact on suicidality was not distinctly superior to other treatments.

Longitudinal studies such as the one by Hafeman et al. [26] provide compelling evidence that Lithium may offer superior outcomes in mood control and reduction in suicidality compared to other mood stabilizers. This longitudinal perspective reinforces Lithium’s role in long-term management, despite the variability in individual responses and adherence issues. The studies by Jairam et al. [40,41] raise concerns about the effectiveness of Lithium in preventing relapse and suicidality, underscoring the need for further research into adherence and treatment optimization. The retrospective studies reviewed, including those by Bashir et al. [42] and Jerrell et al. [29], generally support Lithium safety and efficacy in young patients. However, limitations in sample size and methodological constraints highlight the need for more robust evidence.

Cross-sectional studies reveal interesting associations between Lithium use and reduced self-harming behaviors, though these findings are less conclusive about causality. Studies such as those by Ko et al. [28] and Otuyelu et al. [45] suggest that, while Lithium may be underutilized, its use is associated with lower rates of self-harm and suicide. However, the absence of significant associations in some studies may reflect limitations in study design or sample characteristics. 

The present review highlights a gap in direct evidence regarding Lithium impact on ED, a key factor in suicidality among youths. Although some studies suggest that Lithium may influence ED symptoms, further research is needed to clarify its role and to develop targeted interventions for patients with significant ED. Issues of adherence and treatment duration are critical. As highlighted by longitudinal studies, the effectiveness of Lithium can be compromised by non-compliance or discontinuation. Strategies to improve adherence and monitor treatment response are essential in youth and might include psychoeducation, motivational interviewing, or technology-based interventions. Moreover, the use of extended-release Lithium formulations could also be beneficial. The lack of comprehensive guidelines underscores the need for ongoing research to establish clearer recommendations for Lithium use in youth with BD. Clinicians should consider individual patient factors, including the presence of ED, when prescribing Lithium and evaluate its efficacy regularly.

The available evidence supports Lithium as a potentially effective treatment for reducing suicidal ideation and behaviors in youth with BD. While its benefits are notable, they are tempered by several important considerations. Lithium-established efficacy in mood stabilization translates to potential benefits in reducing suicidality. Nonetheless, its effectiveness in preventing suicides specifically, particularly in younger populations, remains less certain. The safety profile of Lithium is generally favorable, but its use must be carefully monitored, especially in adolescents who may experience different side effects compared to adults. Indeed, some side effects can be particularly concerning in children and adolescents, including thyroid dysfunction and tremors. Moreover, Lithium has a narrow therapeutic index, which is especially challenging in youths who may be more sensitive to fluctuations in blood levels. Due to these aspects, adherence to treatment can be problematic in this age range, and adolescents may be resistant to taking medication that requires such close management. Finally, there is still limited research on the long-term effects of Lithium in youth, since most studies have been conducted in adults, and the long-term safety in young people is not as well established. The long-term use of Lithium is associated with the prevention of mood episodes relapses and the emergence of new symptoms, but this effect has been mainly explored in adult samples. This further contributes to making its use in children and adolescents more cautious, and prescribers often rely on clinical experience and individualized risk–benefit assessments. Nonetheless, the effect of Lithium on ED has yet to be explored, which could help in understanding a possible mechanism underlying its anti-suicidal effectiveness. For this reason, future research aimed to assess the efficacy of Lithium treatment on ED and its mediation over suicidality prevention is welcome.

One of the main limitations of our review is that the methodological quality of most of the included studies was moderate to low, and the overall quality of evidence was poor. Future research should focus on well-designed RCTs with larger and more diverse samples, detailed assessments of ED, and long-term outcomes. This will be particularly relevant since conducting well-designed RCTs is important to establish a stronger causal link between Lithium therapy and reduced suicidality. Additionally, exploring the mechanisms through which Lithium affects suicidality could provide insights into optimizing treatment strategies. Particularly, since evidence is available on the effectiveness of Lithium on both impulsivity and aggression, future studies could address this issue by suggesting that its anti-suicidal properties are mediated by its beneficial effects on such symptom domains. An additional limitation of our review is that it has not been preregistered on an online database for systematic review protocols (e.g., PROSPERO).

## 5. Conclusions

This systematic review reinforces the potential role of Lithium in reducing suicidality among youth with BD, though evidence remains mixed and further research is necessary. The benefits of Lithium in mood stabilization and reduction in suicidal ideation are supported by multiple studies, yet its role in managing ED and ensuring long-term efficacy requires further investigation. Clinicians should continue to use Lithium critically, considering individual patient needs and maintaining a focus on adherence and comprehensive care strategies. In other words, our review recommends the use of Lithium as a potential treatment option for youth with BD and suicidal ideation, but also highlights the importance of careful monitoring and adherence.

## Data Availability

No new data were created.

## References

[1] Pisano S., Pozzi M., Catone G., Scrinzi G., Clementi E., Coppola G., Milone A., Bravaccio C., Santosh P., Masi G. (2017). Putative Mechanisms of Action and Clinical Use of Lithium in Children and Adolescents: A Critical Review. Curr. Neuropharmacol..

[2] Miura T., Noma H., Furukawa T.A., Mitsuyasu H., Tanaka S., Stockton S., Salanti G., Motomura K., Shimano-Katsuki S., Leucht S. (2014). Comparative efficacy and tolerability of pharmacological treatments in the maintenance treatment of bipolar disorder: A systematic review and network meta-analysis. Lancet Psychiatry.

[3] Cipriani A., Hawton K., Stockton S., Geddes J.R. (2013). Lithium in the prevention of suicide in mood disorders: Updated systematic review and meta-analysis. BMJ.

[4] Tondo L., Baldessarini R., Hennen J., Floris G., Silvetti F., Tohen M. (1998). Lithium treatment and risk of suicidal behavior in bipolar disorder patients. J. Clin. Psychiatry.

[5] Tondo L., Baldessarini R.J. (2024). Prevention of suicidal behavior with lithium treatment in patients with recurrent mood disorders. Int. J. Bipolar Disord..

[6] Nabi Z., Stansfeld J., Plöderl M., Wood L., Moncrieff J. (2022). Effects of lithium on suicide and suicidal behaviour: A systematic review and meta-analysis of randomised trials. Epidemiol. Psychiatr. Sci..

[7] Riblet N.B., Shiner B., Young-Xu Y., Watts B.V. (2022). Lithium in the prevention of suicide in adults: Systematic review and meta-analysis of clinical trials. BJPsych Open.

[8] Tueth M.J., Murphy T.K., Evans D.L. (1998). Special considerations: Use of lithium in children, adolescents, and elderly populations. J. Clin. Psychiatry.

[9] Van Meter A., Moreira A.L.R., Youngstrom E. (2019). Updated Meta-Analysis of Epidemiologic Studies of Pediatric Bipolar Disorder. J. Clin. Psychiatry.

[10] Findling R.L., Robb A., McNamara N.K., Pavuluri M.N., Kafantaris V., Scheffer R., Frazier J.A., Rynn M., DelBello M., Kowatch R.A. (2015). Lithium in the Acute Treatment of Bipolar I Disorder: A Double-Blind, Placebo-Controlled Study. Pediatrics.

[11] Findling R.L., Kafantaris V., Pavuluri M., McNamara N.K., Frazier J.A., Sikich L., Kowatch R., Rowles B.M., Clemons T.E., Taylor-Zapata P. (2013). Post-acute effectiveness of lithium in pediatric bipolar I disorder. J. Child Adolesc. Psychopharmacol..

[12] Hauser M., Galling B., Correll C.U. (2013). Suicidal ideation and suicide attempts in children and adolescents with bipolar disorder: A systematic review of prevalence and incidence rates, correlates, and targeted interventions. Bipolar Disord..

[13] Duffy A., Heffer N., Goodday S.M., Weir A., Patten S., Malhi G.S., Cipriani A. (2018). Efficacy and tolerability of lithium for the treatment of acute mania in children with bipolar disorder: A systematic review: A report from the ISBD-IGSLi joint task force on lithium treatment. Bipolar Disord..

[14] Vita G., Nöhles V.B., Ostuzzi G., Barbui C., Tedeschi F., Heuer F.H., Keller A., DelBello M.P., Welge J.A., Blom T.J. (2024). Systematic Review and Network Meta-Analysis: Efficacy and Safety of Antipsychotics vs. Antiepileptics or Lithium for Acute Mania in Children and Adolescents. J. Am. Acad. Child Adolesc. Psychiatry.

[15] Patel N.C., DelBello M.P., Bryan H.S., Adler C.M., Kowatch R.A., Stanford K., Strakowski S.M. (2006). Open-label lithium for the treatment of adolescents with bipolar depression. J. Am. Acad. Child Adolesc. Psychiatry.

[16] Salpekar J.A., Joshi P.T., Axelson D.A., Reinblatt S.P., Yenokyan G., Sanyal A., Walkup J.T., Vitiello B., Luby J.L., Wagner K.D. (2015). Depression and Suicidality Outcomes in the Treatment of Early Age Mania Study. J. Am. Acad. Child Adolesc. Psychiatry.

[17] Scott F., Hampsey E., Gnanapragasam S., Carter B., Marwood L., Taylor R.W., Emre C., Korotkova L., Martín-Dombrowski J., Cleare A.J. (2023). Systematic review and meta-analysis of augmentation and combination treatments for early-stage treatment-resistant depression. J. Psychopharmacol..

[18] Amsterdam J.D., Xu C. (2024). Multi-trial, aggregated, individual participant data mega-analysis of short-term antidepressant versus mood stabilizer monotherapy of bipolar type II major depressive episode. Bipolar Disord..

[19] Dickstein D.P., Towbin K.E., Van Der Veen J.W., Ph D., Rich B.A., Brotman M.A., Knopf L., Onelio L., Pine D.S., Leibenluft E. (2009). of Lithium in Youths with Severe Mood Dysregulation. J. Child Adolesc. Psychopharmacol..

[20] Malone R.P., Delaney M.A., Luebbert J.F., Cater J., Campbell M. (2000). A double-blind placebo-controlled study of lithium in hospitalized aggressive children and adolescents with conduct disorder. Arch. Gen. Psychiatry.

[21] Masi G., Perugi G., Millepiedi S., Mucci M., Pfanner C., Berloffa S., Pari C., Gagliano A., D’Amico F., Akiskal H.S. (2010). Pharmacological response in juvenile bipolar disorder subtypes: A naturalistic retrospective examination. Psychiatry Res..

[22] Leu-Semenescu S., Le Corvec T., Groos E., Lavault S., Golmard J.-L., Arnulf I. (2015). Lithium therapy in Kleine-Levin syndrome: An open-label, controlled study in 130 patients. Neurology.

[23] Berry-Kravis E., Sumis A., Hervey C., Nelson M., Porges S.W., Weng N., Weiler I.J., Greenough W.T. (2008). Open-label treatment trial of lithium to target the underlying defect in fragile X syndrome. J. Dev. Behav. Pediatr..

[24] Masters K.J. (2008). Anti-suicidal and self-harm properties of lithium carbonate. CNS Spectr..

[25] Rosenberg J.M., Salzman C. (2007). Update: New uses for lithium and anticonvulsants. CNS Spectr..

[26] Hafeman D.M., Rooks B., Merranko J., Liao F., Gill M.K., Goldstein T.R., Diler R., Ryan N., Goldstein B.I., Axelson D.A. (2020). Lithium Versus Other Mood-Stabilizing Medications in a Longitudinal Study of Youth Diagnosed with Bipolar Disorder. J. Am. Acad. Child Adolesc. Psychiatry.

[27] Desai Boström A.E., Andersson P., Rask-Andersen M., Jarbin H., Lundberg J., Jokinen J. (2023). Regional clozapine, ECT and lithium usage inversely associated with excess suicide rates in male adolescents. Nat. Commun..

[28] Ko A., Swampillai B., Timmins V., Scavone A., Collinger K., Goldstein B.I. (2014). Clinical characteristics associated with lithium use among adolescents with bipolar disorder. J. Child Adolesc. Psychopharmacol..

[29] Jerrell J.M. (2008). Pharmacotherapy in the community-based treatment of children with bipolar I disorder. Hum. Psychopharmacol..

[30] Tondo L., Baldessarini R.J. (2016). Suicidal Behavior in Mood Disorders: Response to Pharmacological Treatment. Curr. Psychiatry Rep..

[31] Masi G., Pisano S., Sesso G., Mazzullo C., Berloffa S., Fantozzi P., Narzisi A., Placini F., Valente E., Viglione V. (2023). Persistent Non-Suicidal Self-Injury and Suicidality in Referred Adolescents: A Longitudinal Study Exploring the Role of Cyclothymic Temperament. Brain Sci..

[32] Kulacaoglu F., Izci F. (2022). The Effect of Emotional Dysregulation and Impulsivity on Suicidality in Patients with Bipolar Disorder. Psychiatr. Danub..

[33] Fiorillo A., Sampogna G., Albert U., Maina G., Perugi G., Pompili M., Rosso G., Sani G., Tortorella A. (2023). Facts and myths about the use of lithium for bipolar disorder in routine clinical practice: An expert consensus paper. Ann. Gen. Psychiatry.

[34] Kafantaris V., Coletti D.J., Dicker R., Padula G., Kane J.M. (2003). Lithium treatment of acute mania in adolescents: A large open trial. J. Am. Acad. Child Adolesc. Psychiatry.

[35] Kafantaris V., Coletti D.J., Dicker R., Padula G., Pleak R.R., Alvir J.M.J. (2004). Lithium treatment of acute mania in adolescents: A placebo-controlled discontinuation study. J. Am. Acad. Child Adolesc. Psychiatry.

[36] Findling R.L., McNamara N.K., Youngstrom E.A., Stansbrey R., Gracious B.L., Reed M.D., Calabrese J.R. (2005). Double-blind 18-month trial of lithium versus divalproex maintenance treatment in pediatric bipolar disorder. J. Am. Acad. Child Adolesc. Psychiatry.

[37] Pavuluri M.N., Henry D.B., Carbray J.A., Sampson G.A., Naylor M.W., Janicak P.G. (2006). A one-year open-label trial of risperidone augmentation in lithium nonresponder youth with preschool-onset bipolar disorder. J. Child Adolesc. Psychopharmacol..

[38] Geller B., Luby J.L., Joshi P., Wagner K.D., Emslie G., Walkup J.T., Axelson D.A., Bolhofner K., Robb A., Wolf D.V. (2012). A randomized controlled trial of risperidone, lithium, or divalproex sodium for initial treatment of bipolar I disorder, manic or mixed phase, in children and adolescents. Arch. Gen. Psychiatry.

[39] Kowatch R. (2015). Double TEAM: Enhancing Response and Treating Depression in Patients with Bipolar Disorder During a Mixed or Manic Episode. J. Am. Acad. Child Adolesc. Psychiatry.

[40] Jairam R., Srinath S., Girimaji S.C., Seshadri S.P. (2004). A prospective 4-5 year follow-up of juvenile onset bipolar disorder. BIPOLAR Disord..

[41] Jairam R., Srinath S., Reddy Y.C.J., Shashikiran M.G., Girimaji S.C., Seshadri S.P., Subbakrishna D.K. (2003). The index manic episode in juvenile-onset bipolar disorder: The pattern of recovery. Can. J. Psychiatry..

[42] Bashir M., Russell J., Johnson G. (1987). Bipolar affective disorder in adolescence: A 10-year study. Aust. N. Z. J. Psychiatry.

[43] Masi G., Milone A., Scrinzi G., Mucci M., Viglione V., Bruni G., Berloffa S., Pisano S. (2018). Lithium treatment in bipolar adolescents: A follow-up naturalistic study. Neuropsychiatr. Dis. Treat..

[44] Masi G., Berloffa S., Muratori P., Mucci M., Viglione V., Villafranca A., Inguaggiato E., Levantini V., Placini F., Pfanner C. (2020). A Naturalistic Study of Youth Referred to a Tertiary Care Facility for Acute Hypomanic or Manic Episode. Brain Sci..

[45] Otuyelu E., Foldvari A., Szabo E., Sipos V., Edafiogho P., Szucs M., Dome P., Rihmer Z., Sandor J. (2015). Antidepressant drugs and teenage suicide in Hungary: Time trend and seasonality analysis. Int. J. Psychiatry Clin. Pract..

[46] Janiri D., Moccia L., Conte E., Palumbo L., Chieffo D.P.R., Fredda G., Menichincheri R.M., Balbi A., Kotzalidis G.D., Sani G. (2021). Emotional dysregulation, temperament and lifetime suicidal ideation among youths with mood disorders. J. Pers. Med..

[47] Andersson P., Jokinen J., Jarbin H., Lundberg J., Desai Boström A.E. (2023). Association of Bipolar Disorder Diagnosis with Suicide Mortality Rates in Adolescents in Sweden. JAMA Psychiatry.

[48] Patino L.R., Klein C.C., Strawn J.R., Blom T.J., Tallman M.J., Adler C.M., Welge J.A., DelBello M.P. (2021). A Randomized, Double-Blind, Controlled Trial of Lithium Versus Quetiapine for the Treatment of Acute Mania in Youth with Early Course Bipolar Disorder. J. Child Adolesc. Psychopharmacol..

[49] Findling R.L., Kafantaris V., Pavuluri M., McNamara N.K., McClellan J., Frazier J.A., Sikich L., Kowatch R., Lingler J., Faber J. (2011). Dosing strategies for lithium monotherapy in children and adolescents with bipolar I disorder. J. Child Adolesc. Psychopharmacol..

